# How weather triggers the emergence of bats from their subterranean hibernacula

**DOI:** 10.1038/s41598-023-32166-7

**Published:** 2023-04-18

**Authors:** Martin Koch, Julia Manecke, Jan Pablo Burgard, Ralf Münnich, Karl Kugelschafter, Andreas Kiefer, Michael Veith

**Affiliations:** 1 Department of Biogeography, Trier University, Universitätsring 15, 54296 Trier, Germany; 2 Economics and Social Statistics Department, Trier University, Universitätsring 15, 54296 Trier, Germany; 3 ChiroTec, 35102 Lohra, Germany; 4 NABU Rhineland-Palatinate, 55118 Mainz, Germany

**Keywords:** Ecology, Evolution, Animal behaviour

## Abstract

Hibernation is one of the most important behaviours of bats of the temperate zone. During winter, when little food or liquid water is available, hibernation in torpor lowers metabolic costs. However, the timing of emergence from hibernation is crucial for the resumption of the reproductive process in spring. Here, we investigate the spring emergence of six bat species or pairs of bat species of the genera *Myotis* and *Plecotus* at five hibernation sites in Central Europe over 5 years. Using generalized additive Poisson models (GAPMs), we analyze the influence of weather conditions (air and soil temperature, atmospheric pressure, atmospheric pressure trend, rain, wind, and cloud cover) as predictors of bat activity and separate these extrinsic triggers from residual motivation to emerge from hibernation (extrinsic factors not studied; intrinsic motivation). Although bats in a subterranean hibernaculum are more or less cut off from the outside world, all species showed weather dependence, albeit to varying degrees, with air temperature outside the hibernaculum having a significant positive effect in all species. The residual, potentially intrinsic motivation of species to emerge from their hibernacula corresponds to their general ecological adaptation, such as trophic specialization and roosting preferences. It allows the definition of three functional groups (high, medium and low residual activity groups) according to the degree of weather dependence of spring activity. A better knowledge of the interplay of extrinsic triggers and residual motivation (e.g., internal zeitgebers) for spring emergence will help to understand the flexibility of a species to adapt to a changing world.

## Introduction

For small insectivorous bats the physiological and behavioral adaptation to seasons with unfavorable conditions, when food or liquid water is hardly available, is a prerequisite to colonize seasonally fluctuating environments without the need to migrate^1^. They have limited options to accumulate body fat due to restricted storage capacity, but still have high energy requirements as these are proportional to their body mass-to-surface area ratio^2^. Therefore, they can only survive the winter by entering into a series of torpor bouts. Torpor refers to a physiological state in which metabolic processes and other body functions are temporarily reduced to a minimum^3^. To comply, bats choose hibernation sites according to species specific conditions within the site and its surrounding^4^. Such suitable hibernacula are rare, and once selected, site fidelity is high^5–8^. Hence, hibernacula are key habitats for temperate zone bats.

However, bats use their hibernacula not only for hibernation, but also as an important location for inter-individual interactions. Swarming, either in autumn or spring, is the most conspicuous behavior of bats in front of hibernacula. In autumn, it is primarily a mechanism for mating^9–13^, but it also indicates suitable hibernation sites to the juveniles^14,15^. This could prevent their own offspring from choosing unsuitable hibernacula and increases their chance to survive the winter—it thus increases the reproductive fitness of an individual. Since social interaction is usually beneficial within one’s own species^16^, it is important for a bat to synchronize activity with conspecifics. This increases the chance to meet a mating partner in late summer and autumn^17^. Spring-swarming—compared to swarming in late summer and fall—is a rather inconspicuous phenomenon, but it is registered and discussed in year-round activity studies^18,19^. Mating as a reason for swarming in spring has only been described for the brown long-eared bat (*Pecotus auritus*) within the European *Vespertilionidae*^9^.

In general, hibernation is a trade-off between energy preservation and vulnerability. During winter, when prey is not often available to restore fat reserves, torpor reduces metabolic costs, but on the negative side, it prevents rapid response to predators, inhibits immune system functions, and results in slow accumulation of metabolic waste^20^. In addition, the timing of emergence from hibernation is critical for the resumption of the reproductive process. Interestingly, bats emerge from hibernation already before prey is abundant^21^. During this period, female bats need to use social thermoregulation to cope with the metabolic expenses of fertilization and gestation^22–24^. This results in a need for intraspecific synchronization of spring activity for the emergence from hibernacula.

Changing weather conditions may be the most conspicuous potential drivers of synchronized spring emergence, and some studies indicate that temperature and photoperiod changes may serve as a trigger^25^. While active bats outside hibernacula are exposed to changing temperatures and photoperiods, bats inside a subterranean hibernaculum are more or less cut off from outside weather and may not experience any photoperiodic alterations and sometimes not even changes in ambient temperature. Thus, internal triggers such as physiology, hormone levels, circannual zeitgeber, or combinations of them may also play a role in the synchronization of spring emergence from hibernacula. In fact, evidence suggests that circannual rhythms may trigger a bat’s tendency to leave a hibernaculum^26^.

For temperate zone bats, long-term controlled experiments to independently analyze single determinants as potential triggers for spring emergence are nearly impossible to conduct due to the difficulty of keeping bats in a laboratory in near-natural conditions. Therefore, we here adopt a correlative approach to investigate possible weather determinants of spring activity of bat communities at subterranean hibernacula. We studied four species and two pairs of species which are commonly found in subterranean hibernacula across Central Europe^27^: *Myotis bechsteinii, M. daubentonii*, *M. myotis, M. mystacinus/brandtii, M nattereri* and *Plecotus auritus/austriacus*^27^*. M. myotis* and *M. mystacinus/brandtii* often dominate Central European communities of hibernating bats, while *M. daubentonii* is only locally abundant^27^. The two species of *Plecotus* are different in their summer and winter ecology, with *P. auritus* being more specialized to forest habitats than *P. austriacus*^27^. All species differ in where and how they hibernate inside subterranean shelters^28^ and thus also in the way how they potentially perceive extrinsic triggers (e.g. changes of weather conditions outside subterranean hibernacula).

For external (weather) triggers, air temperature (*T*_*A*_), soil temperature (*T*_*S*_) as a proxy for cumulative positive net radiation, atmospheric pressure (*AP*) as well as the atmospheric pressure trend (*APT*), wind speed (*WS*), precipitation rate (*PR*) and cloud coverage (*CC*) are considered. By extracting their influence on the timing of emergence of bats from their hibernacula, the remaining temporal activity pattern should reflect the influence of triggers other than the analyzed weather parameters (e.g. physiology, hormone levels, circannual zeitgeber, weather parameters not captured by our data, or combinations of them; for simplicity, we here subsume them under the term ‘residual triggers’). At the same time, studying the activity of an entire bat community allows identification of species-specific patterns in their activity response to external and residual triggers. We hypothesize that species which are known as typical ‘cave dwellers’ (e.g. *Myotis myotis*) show a less pronounced residual emergence activity than those known to be tree dwellers (e.g. *Myotis nattereri*). Thus, our separation of weather and residual triggers on the emergence from subterranean hibernacula may allow us to define ecological bat functional groups with relation to the timing of emergence.

## Results

Species-specific activity was recorded using a combination of light beam technology and photographs. Bat movements per species/species group were aggregated into hourly activities. Generalized additive Poisson models (GAPM) were applied to analyze the influence of weather parameters (air and soil temperature, atmospheric pressure, atmospheric pressure trend, rain, wind, and cloud cover) on bat activity. In addition, the respective hibernation site itself, the time of sunset and sunrise, and the day of the year were used as predictor variables. The focal species are *Myotis myotis*, *M. nattereri*, *M. daubentonii* and *M. bechsteinii*. In addition, models were inferred for species pairs that cannot be reliably distinguished from photographs: *M. mystacinus*/*M. brandtii* and *Plecotus auritus/P. austriacus*. Details on data collection and processing, as well as the statistical model, can be found in the methodology section.

### Influence of external triggers

**Figure 1:**
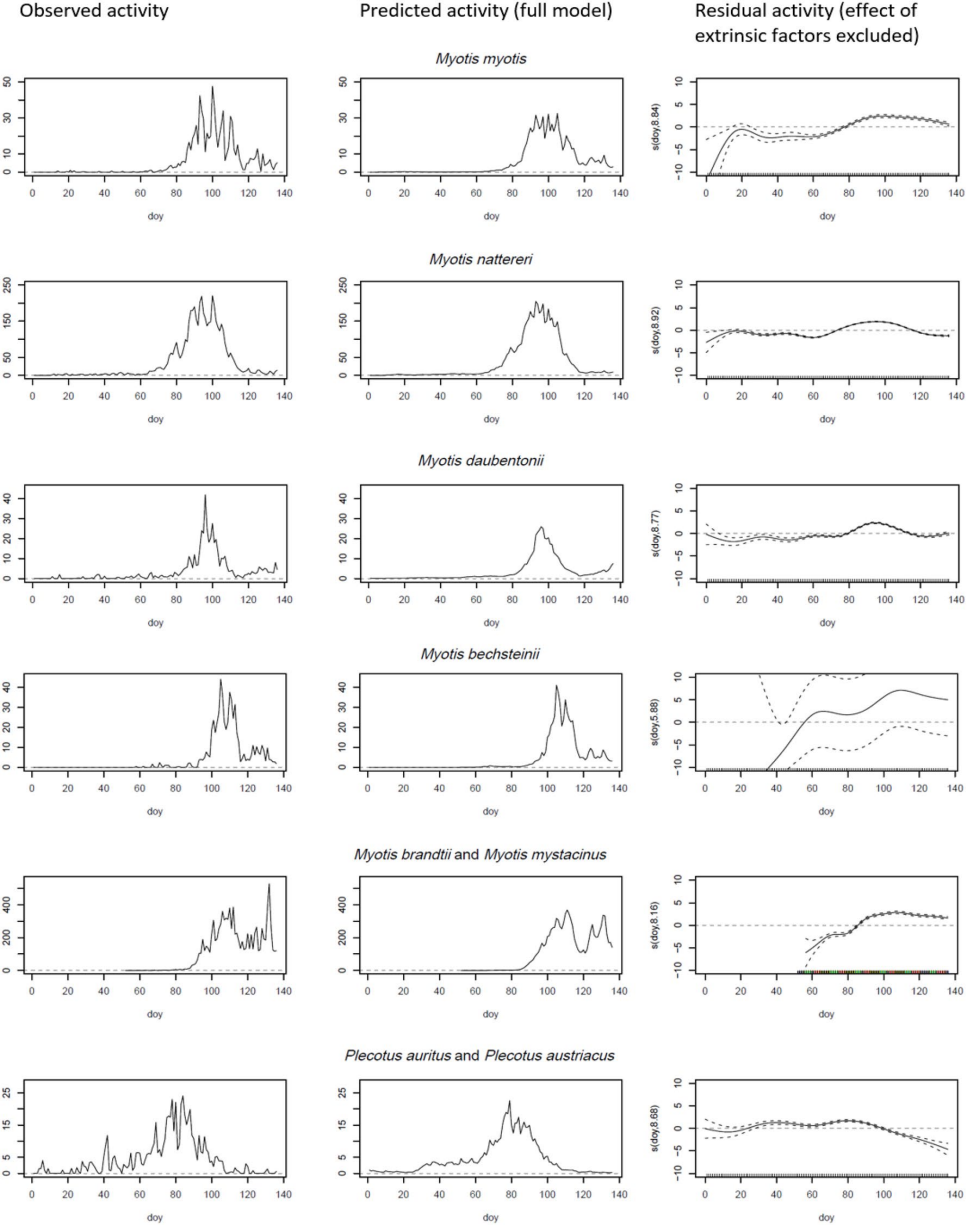
Observed (left column) and predicted spring activity (day 0–136) of bats at five hibernacula, predicted either by the full model (middle column) or by residual triggers alone (right column); horizontal dotted line at value 0 for orientation.

The results of the six GAPMs are summarized in Table 1. They include the parametric coefficients and significance of the linear effects in sections 1a and 1b. These were divided into the factor variable for the respective site (section 1a) and the seven weather-related variables (section 1b). The reference category of the site factor (hibernaculum) was the hibernaculum Mayen-Mauerstollen. This factor accounted for site-specific differences in the frequency of the species. Thus, a negative coefficient for a site implies that a reduced activity of a species was to be expected at the respective site compared to the reference site Mayen-Mauerstollen. Most species were less active at all sites compared to the reference site, except for *M. daubentonii* at Fischendorf and Rabenstein and *Plecotus* spec. at Rabenstein. As the regression splines are nonlinear effects, their coefficients were not comparable to the linear coefficients displayed. Therefore, only the approximate significance of the smooth terms were included in Table 1, section c. A quantification of the effect of the spline, i.e., the smoothing curve that fitted the effect of the variable ‘day of year’ (*DOY*), is depicted in the right column of Fig. 1. The explained deviance (see Table 1, section 1d) defines the proportion of the deviation from zero that is explained by the model. It ranges between 59.1% for *M. daubentonii* and *P. auritus/austriacus* and 84.8% for *M. nattereri.* The total number of observations (= number of hours recorded after excluding hours with missing covariates) depends on the respective hibernation sites that were considered for the model estimation based on the occurrence of the respective species.

**Table 1:** Characterization of the six GAPMs.

		*Myotis myotis*	*Myotis nattereri*	*Myotis daubentonii*	*Myotis bechsteinii*	*Myotis brandtii/ Myotis mystacinus*	*Plecotus auritus/ Plecotus austriacus*
		(GAPM 1)	(GAPM 2)	(GAPM 3)	(GAPM 4)	(GAPM 5)	(GAPM 6)
1a	Intercept	− 13.609***	− 9.474***	− 27.781***	1.456	− 21.125***	4.425*
		(1.897)	(0.740)	(2.368)	(4.795)	(1.074)	(2.008)
	Fischendorf^a^	− 2.542***	− 1.267***	0.375***			− 0.894***
		(0.065)	(0.018)	(0.042)			(0.068)
	Kaub^a^	− 2.436***	− 7.760***	− 2.412***	− 2.462***		− 2.520***
		(0.062)	(0.354)	(0.111)	(0.082)		(0.092)
	Neuenkirchen-Sulzbach^a^	− 1.985***	− 1.838***	− 2.187***	− 1.612***		0.074
		(0.058)	(0.023)	(0.109)	(0.084)		(0.044)
	Rabenstein^a^	− 4.015***	− 1.641***	0.508***			0.288***
		(0.125)	(0.018)	(0.039)			(0.037)
1b	Soil temperature (*T*_*S*_)	− 0.055***	0.001	0.074***	0.039*	0.117***	0.056***
		(0.010)	(0.004)	(0.010)	(0.018)	(0.006)	(0.012)
	Air temperature (*T*_*A*_)	0.173***	0.120***	0.057***	0.154***	0.066***	0.104***
		(0.006)	(0.002)	(0.007)	(0.009)	(0.003)	(0.007)
	Air pressure (*AT*)	0.011***	0.009***	0.023***	− 0.010***	0.018***	− 0.008***
		(0.002)	(0.001)	(0.002)	(0.003)	(0.001)	(0.002)
	Air pressure trend (*ATP*)	0.015	0.035***	0.026	0.042	0.0749***	− 0.071***
		(0.018)	(0.007)	(0.022)	(0.029)	(0.010)	(0.019)
	Wind speed (*WS*)	− 0.126***	− 0.063***	− 0.120***	− 0.074***	0.007	− 0.090***
		(0.010)	(0.003)	(0.010)	(0.016)	(0.005)	(0.009)
	Precipitation rate (*PR*)	− 0.367***	0.006	0.175***	− 0.463***	− 0.033	− 0.360***
		(0.084)	(0.024)	(0.048)	(0.114)	(0.029)	(0.083)
	Cloud coverage (*CC*)	− 0.005	0.001	0.024***	− 0.018**	− 0.021***	− 0.043***
		(0.005)	(0.002)	(0.005)	(0.006)	(0.002)	(0.005)
1c	Sun Time (*ST*)	***	***	***	***	***	***
	Day of Year (*DOY*)	***	***	***	***	***	***
1d	*N* Observations	20,760	20,760	20,760	13,275	5050	20,760
	Deviance explained	0.754	0.848	0.591	0.765	0.845	0.591

The sections address different parts of the respective models.

^a^Reference site: Mayen Mauerstollen.

*p < 0.05, **p < 0.01, ***p < 0.001.

Soil temperature (*T*_*S*_) had a significant positive effect on the activity of *Myotis daubentonii*, *Myotis bechsteinii* and the species pairs *Myotis mystacinus/brandtii* and *Plecotus auritus/austriacus*. In contrast, with an increase of *T*_*S*_ the activity of *Myotis myotis* was reduced significantly. No significant effect was observed for *Myotis nattereri.* For air temperature (*T*_*A*_) a significant positive effect was measured on all species. High air pressure had a significant positive effect on the activity of *M. myotis*, *M. nattereri*, *M. daubentonii*, and *M. mystacinus/brandtii*. Activity of *M. bechsteinii* and the *Plecotus* species were significantly decreasing with increasing *AP*. To consider the direction in change of the variable *AP* we included *APT* as variable capturing the air pressure trend. An increasing air pressure trend had a positive effect on the activity of *M. nattereri* and *M. mystacinus/brandtii,* which is highly significant at the 0.1% level (*p* < 0.001). In contrast, an increasing *ATP* had a significant negative effect on the activity of *P. auritus/austriacus*. Wind speed (*WS*) has a significant negative effect on all observed species but *M. mystacinus/brandtii*. Rain (*PR*) in the hour of observation had a significant negative effect on the activity of *M. myotis*, *M. bechsteinii* and the *Plecotus* species, but a significant adverse effect on *M. daubentonii*. Cloud coverage (*CC*) had a significant positive effect on *M. daubentonii.* However, it has a negative effect on *M. bechsteinii, M. mystacinus/brandtii* and *P. auritus/austriacus.* The smooth terms of all models were significant at the 0.1% level according to a χ^2^ test (see Table 1, Section c). This included the variables *ST* and *DOY*, which indicated that, apart from extrinsic weather-related factors, the observed species’ motivation to leave the hibernacula was additionally influenced by residual triggers.

### Influence of residual triggers

**Figure 2:**
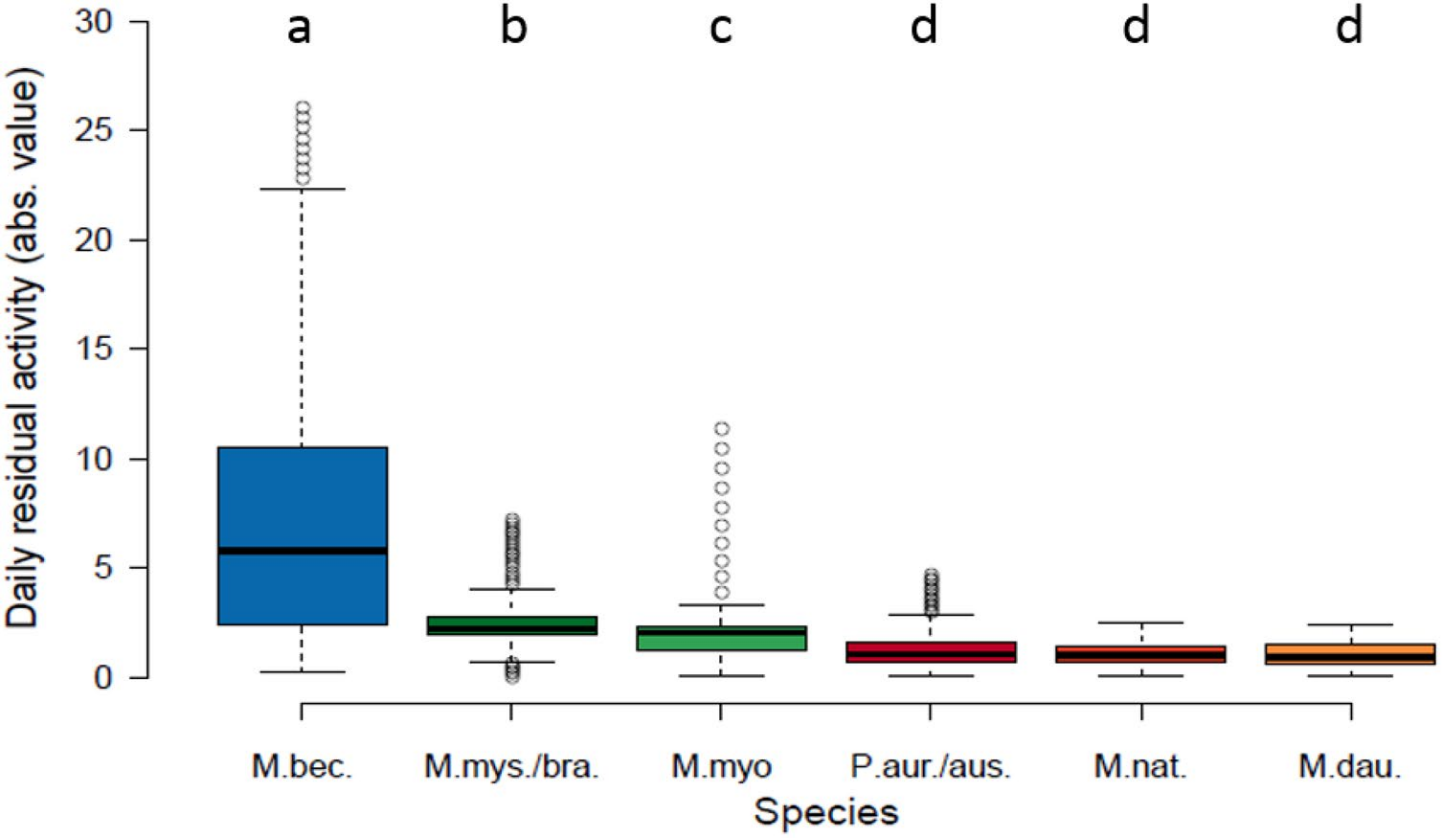
Species in order of decreasing median of absolute values of residual activity from zero (= increasing dependency from ambient weather). M.bec. = *Myotis bechsteinii*, M.mys./bra. = *Myotis mystacinus/brandtii*,M.myo. = *Myotis myotis*, P.aur./aust. = *Plecotus auritus/austriacus*, M.nat. = *Myotis nattereri*, M.dau. = *Myotis daubentonii*; The letters indicate species groups of non significant pairwise post-hoc tests with Bonferroni correction for multiple tests (*p* < 0.05).

After eliminating the effect of the analyzed extrinsic factors, the residual activity allows for a comparison among species between day 0 (January 1st) and day 136 (15th/16th of May). The right column in Fig. 1 shows the predicted activity that has been modelled in dependence of *DOY* alone, regardless of the weather covariates. In doing so, we try to identify day-of-year-dependent activities over the course of advanced winter and spring season that cannot be explained by the observed extrinsic triggers. Negative values indicate a low residual motivation, even when the weather conditions are favorable. In contrast, positive values stand for an increase in activity even if the weather conditions are less ideal. Values around zero indicate that activity is mostly triggered by external factors. Therefore, the more residually motivated activity deviates from zero during spring, the less dependent a species is from the weather outside the hibernacula. *M. bechsteinii* shows the highest residual activity and thus is by far the least weather dependent species (Fig. 2). The group of species whose activity is least triggered by residual factors comprises *M. daubentonii*, *M. nattereri* and the two *Plecotus* species. *M. mystacinus/brandtii* and *M. myotis*, although significantly different from each other in their residual activity, show an intermediate dependency from the weather outside their hibernacula.

### Timing and synchronization of emergence

**Figure 3:**
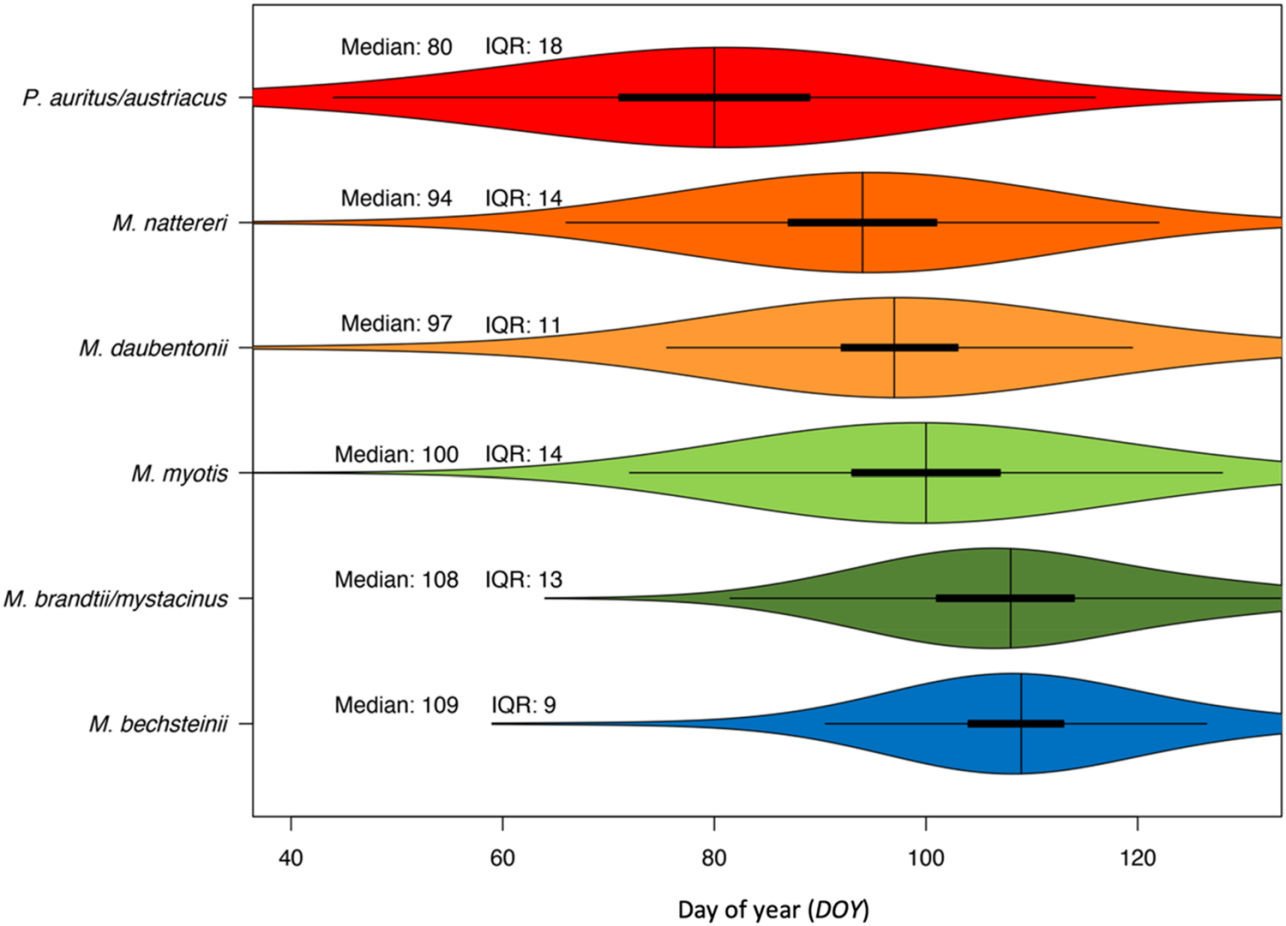
Temporal duration and median of increased spring activity of bat species across five hibernacula; the interquartile range (IQR) is an indicator for the degree of synchronization (without outliers); colours according to Fig. 2.

A comparison between observed and predicted activity patterns (left and middle columns in Fig. 1) shows that the considered model covariates seem to shape overall spring activity of bats at hibernacula. When comparing the temporal activity pattern among species as predicted by the models (Fig. 3), it becomes obvious that activity peaks earliest in *Plecotus* spec. (*DOY* 80), followed by *M. nattereri, M. daubentonii* and *M. myotis*. M. mystacinus/brandtii and Myotis bechsteinii are the last to show peak activity in spring (*DOY* 108 and *DOY* 109 respectively). The interquartile range (IQR) indicates the numbers of days within which the mean 50% of the activity takes place and may serve as a proxy for activity-synchronization within a species. It shows that spring activity at the analyzed hibernacula is highly synchronized in *M. bechsteinii* (IQR = 9), while it is less synchronized in other species, with the longest duration of spring activity being observed in *P. auritus/austriacus* (*IQR* = 18).

**Figure 4:**
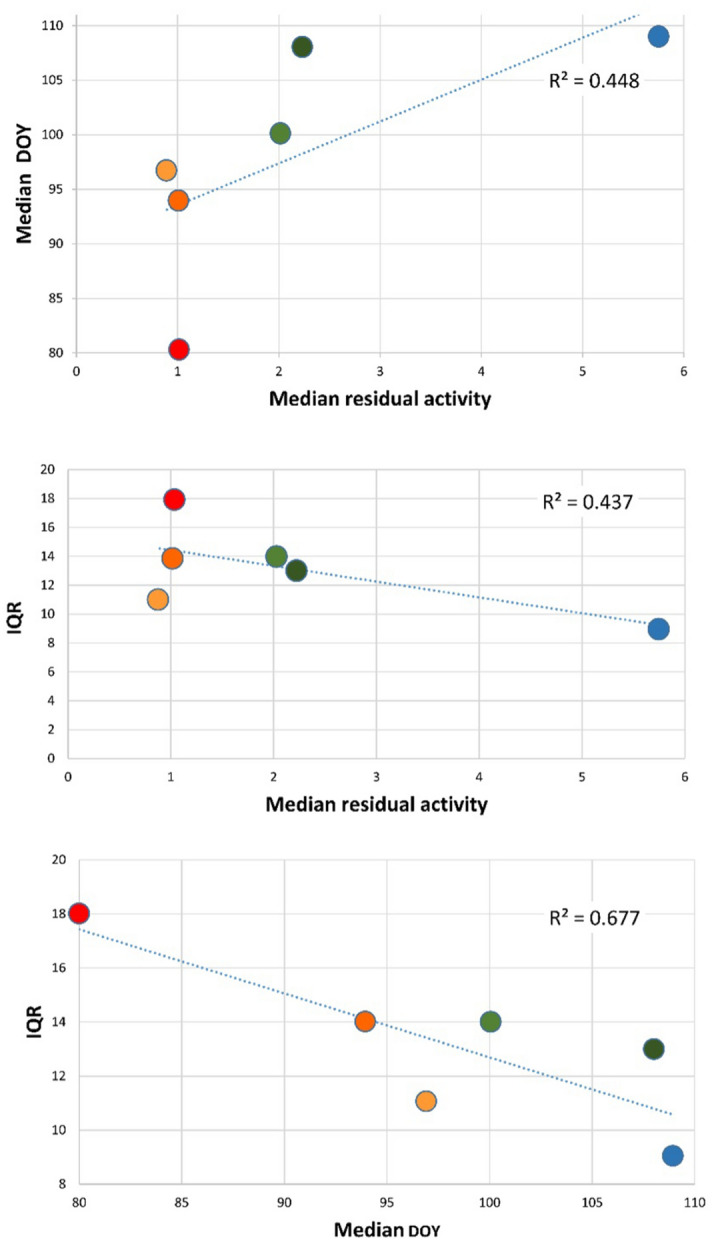
Relation between mean residual activity, median DOY of emergence and the interquartile range (IQR) of emergence activity; colors according to Fig. 2.

Timing (DOY) and synchronization (IQR) of emergence activity correlate with the mean residual activity the species studied, however not significantly (*p* > 0.05); nevertheless, ca. 45% of the variance of mean DOY of are explained by the mean residual activity in both cases. In contrast, ca. 68% of the variance of the IQR are explained by variation in the timing of emergence (mean DOY) (*p* < 0.05) (Fig. 4).

## Discussion

The inclusion of observations from five different sites, albeit with data gaps, introduces variability due to possible local determinants of activity. Such site specificity may be the landscape in which the hibernaculum is located, microclimatic characteristics, geophysical structures, as well as the local density of further suitable hibernacula. In addition, its functional integration into associated local and regional bat communities may vary even within a species^3,4,29,30^. However, by incorporating data from multiple sites with diverse bat communities, we were able to identify general triggers of species-specific spring activity of bats that may be transferable to other Central European bat hibernacula.

Perception of ambient weather is not a problem for bats that hibernate in tree roosts or rock crevices. Underground, however, e.g. deep in caves and abandoned mines, the animals are largely cut off from direct perception of the weather. Therefore, it is first of all astonishing that the different weather parameters can have an impact on the emergence of the species we studied. Air temperature (*T*_*A*_) represents a weather parameter that bats usually can react to immediately when they perceive it. In our study, all species show a significant increase in activity with increasing *T*_*A*_ in spring. However, inside a hibernaculum, away from the entrance, temperature becomes increasingly stable and less affected by outside *T*_*A*_^31^. In contrast, soil is a much more inert mass and does not respond to daily fluctuations in net radiation. As a result, changes in soil temperature (*T*_*S*_) may better represent a trend in daily net radiation over time^32,33^, indicating e.g. the onset of spring. Bats do not directly experience soil temperature. But a continuous inversion of the temperature gradient from outside temperatures to inside temperatures changes the air flow within a hibernaculum^31^ and may provide bats deep underground with information from the outside world. In addition, soil, particularly forest soil with its leaf litter, harbors enormous quantities of insect larvae and pupae, providing an important source of future insect prey for temperate zone bats^34^. For *M. nattereri*, no significant change in activity with change in *T*_*S*_ was detectable in our study; this species is known to do foraging bouts during the winter^35^. *M. myotis* may have already left the hibernaculum when *T*_*S*_ increases, this can explain why also this species does not react significantly to *T*_*S*_.

Atmospheric pressure (*AP*) and atmospheric pressure trend (*APT*) are the only environmental parameters that change more or less simultaneously at the surface and deep underground^31^. Bats have been shown to track atmospheric pressure and respond to its changes by changing their metabolic rate^36^. In spring, an increase in atmospheric pressure indicates warmer temperatures and a seasonal change due to longer days and a higher angle of the sun’s orbit^37^. *M. bechsteinii* and *P. auritus/austriacus* show a negative correlation between activity and *AP*. Since *P. auritus* may be the dominant species of the species pair *P. auritus/austriacus*, and since this species mates in spring^9^, activity in spring is biased to mating activity during unfavorable foraging conditions at the sheltered entrances of the hibernacula. We believe that the counter-intuitive correlation between an increasing activity of *M. bechsteinii* and decreasing atmospheric pressure is an artefact, because non reproductive females and males use the sheltered hibernaculum in late spring for short term torpor to endure unfavorable weather conditions outside the cave. At the same time, increased wind speed is often associated with decreasing temperatures and increasing precipitation rate (*PR*)^37^. In general, wet, cold and windy weather increases thermoregulatory costs for a bat^38^. In addition, rain also inhibits insect flight^39^, lowering a bat’s potential energy gain through foraging. Suppression of swarming activity by precipitation and high wind speed therefore biologically makes sense and has been shown previously^19,40^.

The immediate effect of clouds, measured in our study by cloud cover (*CC*), corresponds to a reduction in brightness. There is only little information on how natural light reduction by clouds affects bat activity, but even a short reduction of brightness by a lunar eclipse increased bat activity^41^, and illumination in general may interrupt habitat connectivity for bats on a landscape level^42^. In addition, *CC* often acts in concert with temperature, precipitation, wind speed, and even atmospheric pressure. Since such covariation was extracted in our models, *CC* indeed has a significant and independent positive effect on the activity of *M. daubentonii.* On *P. auritus/austriacus, M. mystacinus/brandtii* and *M. bechsteinii*, this effect is significantly negative. The genus *Myotis* in general is presumed to be sensitive to light at night^43–45^.

The weather parameters we analyzed do not predict the emergence from the hibernacula in the same way for all species, as shown by our species-specific GAPMs. Rather, different species show a different sensitivity to weather parameters at different times in spring. However, our data clearly show that emergence from hibernation is triggered not only by the ambient weather parameters studied by us, but also by residual factors. By extracting the effects of weather on activity, we were able to quantify this species specific residual activity, which may serve to distinguish three functional groups (we abstain from using the term ‘guild’, since in bat ecology it is most often used to define groups of bats with similar foraging strategies^46^): high residual activity group (HRA; *M. bechsteinii*), medium residual activity group (MRA; *M. mystacinus brandtii, M. myotis*), and low residual activity group (LRA; *P. auritus/austriacus, M. nattereri, M. daubentonii*).

Interestingly, the three functional groups as defined by the median residual activity (Fig. 4) also differ in characteristics of emergence from wintering grounds. LRA species emerge earlier and less synchronously in spring (higher interquartile range, IQR) than MRA and the single HRA species. Only in the IQR is there a significant overlap between the MRA and LRA groups. Within the LRA functional group, emergence characteristics do not always vary equally among *P. auritus/austriacus*, *M. nattereri*, and *M. daubentonii*, while in the MRA group the species pair *M. mystacinus/brandtii* always shows more similarity to *M. bechsteinii* (HRA functional group) than *M. myotis*. It is obvious that the two species of the MRA functional group respond similarly to the weather parameters only in *T*_*A*_ and *AT*. Within the LRA functional group, *P. auritus/austriacus* differ from the two other species in their response to weather parameters. *M. nattereri* and *M. daubentonii* show an almost identical response to weather parameters.

The discussed extrinsic factors which may trigger the emergence of bats from a hibernaculum may affect the microclimate in a hibernaculum and can be recognized by the bats^31,47,48^. The factors *Ts* and *APT* represent prolonged changes in the weather conditions. However, as shown in Fig. 1, not all bat species react simultaneously to the same weather conditions. We assume that there is an underlying species specific motivation to emerge from the hibernaculum. Species-specific differences should therefore be consistent with the overall life history of a species, as is the intrinsic motivation for leaving the hibernaculum.

*Myotis bechsteinii* stays longest in its hibernacula. Bechstein’s bat is a distinct forest species with a preference for deciduous forest. Its main prey is small Lepidoptera and insectivorous Orthoptera^49^. Many of the Lepidoptera larvae feed on leaves, and the Orthoptera feed on these Lepidoptera larvae as well as on aphids. Therefore, the main prey taxa of *M. bechsteinii* do not appear until herbaceous plants and foliage has come into leaf and provide food for the preferred prey taxa. This is realized late in spring, which may explain why the species emerges latest of all studied species. As a forest specialist, *M. bechsteinii* chooses tree cavities over buildings as maternity roosts. Tree cavities offer a tempered microclimate, with not only milder temperatures during hot days and warmer temperatures during cold nights in the roost^50^, but also a delayed warming of the shelters in spring^51^. Therefore, to avoid being trapped in cold roosts and thereby running the risk of increased metabolic costs during fetal development, *M. bechsteinii* stays longer in the hibernaculum and occupies tree holes later in the year. The high degree of synchronization of emergence from hibernacula increases the probability of encountering conspecifics at summer roosts right after arriving and offers the immediate benefit of social thermoregulation that can compensate for unfavorable microclimatic conditions in tree-roosts in late spring^24^. So *M. bechsteinii* waits longest to leave the hibernacula and synchronizes with conspecifics, possibly to benefit from social thermoregulation during sporadic unfavorable weather conditions in late spring.

*Myotis mystacinus* and *M. brandtii* are ecologically similar in terms of site selection and trophic niche^27^. Summer roosts are found in crevices in trees, under loose bark, as well as in buildings. Such roosts provide shelter and warm up as the ambient temperature increases, but they do not prevent chilling out during cold weather conditions. These adverse conditions increase the energetic costs of not interrupting fetal development. These costs can be prevented by social thermoregulation. Our data suggest that *M. mystacinus/brandtii* leave their hibernacula on average almost as late as *M. bechsteinii*, although less synchronized. In spring, their motivation increases until *DOY* = 100 and then constantly remains above zero. Thus, the species pair is more dependent on extrinsic factors than *M. bechsteinii*, with temperature, atmospheric pressure, atmospheric pressure trend, and cloud cover significantly influencing their activity. So maybe due to temperature-volatile roosts, these two species leave hibernacula comparatively late. However, it should not be ignored that two species, even if ecologically similar, are analyzed together, which may mask species-specific and potentially significant effects of the parameters analyzed (e.g., the lack of emergence).

*Myotis myotis* is a gleaning bat species, specialized to feed on epigean arthropods, mainly beetles. It is the only species for which decreasing ground temperature and flight activity at the entrances to its hibernacula are negatively correlated. This suggests that the timing of emergence is less dependent on prey availability and more residually motivated. *M. myotis* leaves its hibernacula at a similar time and with a similar degree of synchronization as other species such as *M. nattereri* and *M. daubentonii*. However, it is triggered much more by residual motivation than in the latter two. This can be attributed to the fact that the greater mouse-eared bat lives in larger aggregations than any other Central European bat species. They form maternity colonies of up to 5000 females in attics of buildings. This type of roost provides favorable conditions in spring as they warm up quickly. The cold weather after leaving the hibernacula can be endured by social thermoregulation. This advantage of individuals clustering together can also be achieved when a small part of the colony has already emerged from the hibernacula^22^. The possibility to stay homoeothermic with little energetic investment ensures continuous fetal development and an early birth of the young. This correlates with increased juvenile survival and reduces selection pressure on *M. myotis* to live in smaller associations or to choose colder roosts^52^. *Myotis myotis* may trust the advantages of warm roosts and thermoregulation and by that reduces dependency on favorable weather conditions in spring.

*Myotis nattereri* and *Myotis daubentonii* show a similar timing and synchronization when leaving the hibernacula. They choose a similar type of maternity roost (tree cavities and buildings) but differ in foraging strategy and preferred prey. *M. daubentonii* is specialized in catching aquatic insects from water surfaces^27,53^, while *M. nattereri* feeds on diurnal and often non-flying arthropods collected from vegetation surfaces^54^. Both species are of similar size and weight and form small to medium-sized nursery colonies in trees and buildings. It is suggested that small bat species may need to wake up more frequently to drink during hibernation due to an unfavorable surface-to-volume ratio^55^. Once an individual bat is active, it may have a high motivation to leave the hibernaculum to forage^56^ and relocate to summer habitats.

The activity of *Plecotus auritus/austriacus* extends over the longest period of all observed bat species. This can be explained by the specific reproductive cycle of *Plecotus auritus*, which differs from that of other bat species. *Plecotus auritus* shows extensive swarming and reproductive behavior in spring, right after hibernation^9,57^. Male activity begins early in the year, and the peak of activity is not necessarily associated with the transition from the hibernaculum to the summer roost. It is possible that parts of the species’ spring activity is correlated with mating. *Plecotus auritus/austriacus* occasionally form small maternity colonies in thermally beneficial roost like buildings and tree cavities. Even in winter, prey is available, albeit in low abundance, and is exploited by *Plecotus auritus*^58,59^. The sensitivity to weather with increased prey availability in winter and the spontaneous response to exploit this winter food has been found for *Plecotus auritus* from different parts of its range^60^. So food availability and mating season in late winter increases activity over a long period of time and reduces synchronization. A permanent activity level enables the species group to react to spontaneous acceptable weather conditions to emerge from hibernacula.

All species included in our analyses show a distinct pattern of long inactive (torpor) and shorter active periods during winter. We assume that the onset and peak of activity in spring marks the time of arousal and leaving the hibernaculum. Our data clearly show that awakening is not fully synchronized within a species, but species-specific peaks of activity can be distinguished. For the North American species *Myotis lucifugus* it is shown that synchronization of arousal increases as winter advances^61^. But even less than half of the bats were fully synchronized, with overlapping arousals. A species-specific hormonal regime that regulates the duration of hibernation and arousal^62–64^ may optimize the timing at which a bat species leaves the hibernaculum. This optimal timing may depend more on roost choice and social thermoregulation than on prey availability.

The timing of arousal after hibernation is part of a bat’s circannual rhythm, which has been demonstrated for many hibernating animal species and is of particular importance in perennial organisms^65,66^. Although the underlying mechanisms for circannual rhythms are still not well understood^67^, our study sheds new light on the interplay of weather and residual triggers for the synchronization of species specific emergence from their hibernacula.

## Methods

### Data collection

**Figure 5:**
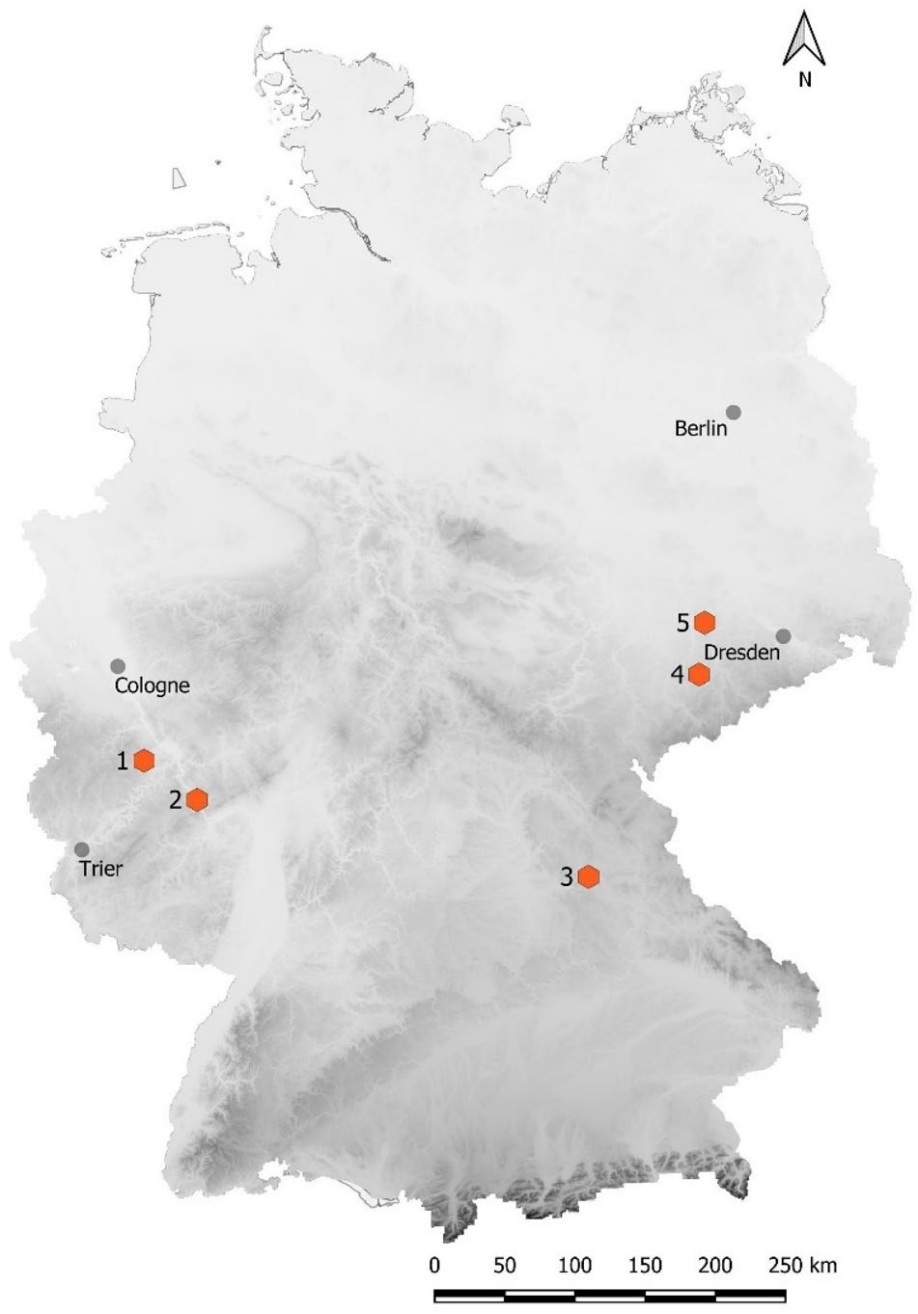
Location of the observed hibernacula in Germany.

We collected data at five hibernacula: (1) Mayen-Mauerstollen, (2) Kaub-Rennleiterstollen, (3) Neukirchen-Sulzbach, (4) Rabenstein (Felsendome Chemnitz) and (5) Fischendorf (Fig. 5). All are abandoned mines of varying size and geophysical characteristics. The temporal coverage of data collection varied across sites due to local constraints of automated recording devices; however, in total our data cover spring activity of bats at the entrances of subterranean bat hibernacula between 2013 and 2017.

To record animals entering and leaving the hibernacula, infrared (IR) light beam technology from ChiroTech (Lohra, Germany) was used; a double row of IR light beams allows the discrimination of bats entering or leaving the hibernaculum. The system was combined with two digital cameras (Lumix DMC-G5K, Panasonic Inc.) each with an external flash to capture a photo of every animal that interrupted the light beams. One camera was placed inside the hibernaculum, facing incoming animals, and one camera was placed outside, facing exiting animals^68^. Recent studies show that there are no negative effects of the flashes on bat activity^69^. Only photos that clearly show bats entering or exiting the hibernaculum were considered for statistical analyses. Species identification on the photos was done manually according to current identification key^27,70^. Data analysis was performed using the software BatLabel^71^. We only included species in the analyses where the number of photographs over the entire 5-year period was considered high enough to conduct a sufficiently meaningful species-specific analysis (Table 2).

**Table 2:** Number of photographs per included species per site.

Species / Hibernaculum	Mayen-Mauerstollen (1)	Kaub-Rennleiterstollen (2)	Neukirchen-Sulzbach (3)	Rabenstein (4)	Fischendorf (5)	Total
*M. mystacinus / brandtii*	46,775	32	3	31	83	**46,924**
*M. bechsteinii*	5714	719	412	–	–	**6845**
*M. nattereri*	76,287	250	5084	8328	14,889	**104,838**
*P. auritus/austriacus*	4387	645	2481	2943	1578	**12,034**
*M. myotis*	14,517	1074	806	279	883	**17,559**
*M. daubentonii*	3858	582	311	3254	3888	**11,893**
Total	**151,538**	**3302**	**9097**	**14,835**	**21,321**	**200,093**

### Data processing

A data set with evaluated photos is available for each observed hibernaculum. For each photo, the data set contains information on the respective time of recording, the respective identified species and whether an entry or an exit was photographed. In the photo datasets, two main activity periods are recognizable for all species in spring and autumn, with a flattening of activity in summer. In order to focus on the triggers that stimulate bat activity in the second half of winter and spring, only part of the photo data set up to day 136 was further analyzed (January 1st to May 15th/16th). The decision for the 136th day as the last day of the observation period is based on statistical analyses of the flight behavior of the observed species throughout the year. Day 136 is the day on which the vast majority of the activity during the main activity period in spring has taken place for all analyzed species and a clear flattening of activity can be observed.

To measure and quantify the level of activity over time, an hourly level data set was constructed. Therefore, the numbers of incoming and outgoing bats per hour were aggregated per species. To mitigate the effect of short-term fluctuations in hourly activity, e.g. the presence of a predator, the number of incoming and outgoing bats per hour each were smoothed by a moving average over three hours. This hourly dataset for each site was supplemented by the hourly weather data of the respective nearest weather stations of the German Meteorological Service (DWD) (Table 3). They cover weather parameters that are known to either affect the flight activity of bats directly or the activity of their prey: air temperature in (*T*_*A*_ in °C), cloud cover in 1/8 (*CC*), precipitation (*PR* in mm), air pressure at mean sea level (*AP* in hPA), soil temperature in 20 cm depth (*T*_*S*_ in °C) and mean wind speed (*WS* in m/s). Furthermore, we added an air pressure trend variable (*APT*) by calculating the air pressure difference between the subsequent and the preceding hour as *APT*_t_ = *AP*_t+1_-*AP*_t-1_. As sunrise and sunset are assumed to influence activity of bats, the time-variable was transformed into a sun-related time. This ensured that the photoperiod was included in the analyses. The ’sun time’ (*ST*) can take values between -1 and + 1, with the hour of sunset assigned the value 0 and sunrise assigned the values -1 and + 1, respectively. In addition, the variable ‘day of year’ (*DOY*) represents the number of the respective day in each year.

**Table 3:** Weather stations considered for each hibernaculum and distance between weather stations and hibernaculum.

Hibernaculum	DWD Weather station (DWD Station ID)	Distance to site (km)
Mayen-Mauerstollen	Nürburg-Barweiler (03660)	26
Kaub-Rennleiterstollen	Geisenheim (01580)	17
Neukirchen-Sulzbach	Pommelsbrunn-Mittelburg (03975)	14
	Kümmersbrück (02773)	24
Rabenstein	Chemnitz (00853)	5
Fischendorf	Geringswalde-Altgeringswalde (00131)	9
	Oschatz (03811)	19

In order to derive a response variable, both the smoothed number of entering bats and the smoothed number exiting bats are compared. The smaller of the two aggregates is defined as the response variable for the respective hour. This helps to mitigate the effect of overall fluctuations resulting from general outflow movements in connection with emergence from hibernation.

### Statistical model

The hourly data of all five study sites were included in the model to predict the hourly number of activity movements as a function of the weather variables and time-specific predictors. Since a simple linear statistical model based on a Gaussian density curve can produce negative values (= negative activity), it is not suitable for modelling bat spring activity. An alternative is the use of a Poisson distribution within a generalized linear model (GLM), which extends simple linear statistical models in order to comply with non-normal distributions of response variables. This avoids negative realizations from the model, which is one reason why Poisson distributions are especially suitable for count data. Bat activity may be, among others, a function of *DOY*. However, *DOY* has a recurrent effect in terms of an annual cycle. Therefore, it can be assumed that the activity is a periodic function of *DOY*, which can be modelled using cyclic regression splines^72^. We here apply a cyclic cubic regression spline. It belongs to the broader class of non-parametric smoothing methods, which are useful when the relationship between the dependent variables and the explanatory variables is non-linear^73^. For the classic cubic regression spline, the respective explanatory variable is divided into a specified number of intervals. Then, a cubic polynomial is fitted in each of these intervals. The resulting fitted values per interval are connected in order to construct a smoothing curve^74^. Under the cyclic extension of the cubic regression spline, the ends of the curve are also matched up to the second derivative^75^. The dependence of the activity on ‘sun time’ (*ST*) is also modelled using a cyclic cubic regression spline, while this explanatory variable has a recurrent effect in terms of a daily cycle. The inclusion of a cyclical variable in a GLM, which is based on a linear relationship between the predictors and the dependent variables, is not trivial. Also, the weather variables are still to be included as linear predictors. One alternative to GLM is the generalized additive model (GAM). Within a semi-parametric GAM, parametrically fitted covariates, such as linear predictors for the weather variables, can be additively combined with nonparametric smoothing functions, such as the cyclic cubic splines^73^. In order to comply with non-normal response distributions, we use a Poisson GAM (GAPM) for the analyses of the influence of covariates on the hourly bat activity. The mathematical formula of our data using the number of bat movements per hour $k$ as our response variable $Y_{k}$ is given by

$$\begin{aligned} Y_{k} & \sim Poisson\left( {\mu_{k} } \right)\;{\text{and}}\;E\left[ {Y_{k} } \right] = \mu_{k} \;{\text{with}}\;log\left( {\mu_{k} } \right) = g\left( {x_{k} } \right),{\text{where}} \\ g\left( {x_{k} } \right) & = \alpha + site_{k} + \beta_{1} T_{S,k} + \beta_{2} T_{A,k} + \beta_{3} AP_{k} + \beta_{4} APT_{k} + \beta_{5} WS_{k} + \beta_{6} PR_{k} + \beta_{7} CC_{k} \\ & \quad + f_{1} \left( {ST_{k} } \right) + f_{2} \left( {DOY_{k} } \right) + f_{3} \left( {ST_{k} , DOY_{k} } \right) \\ \end{aligned}$$

The hibernacula are included as factor variable (site) to account for site-specific differences. We considered and tested both the inclusion of the respective site and the respective year as factor variables in the model. While site had a considerable effect on the improvement of the model quality due to the significantly different occurrence of the species at the sites, this effect was absent when the year was added to the model. It should also be noted that adding year would also require an interaction with the variable site. This is due to the fact that the occurrence of a species is very unlikely to develop uniformly across all sites over the years. This added complexity but lack of improvement in model quality and led us decide to not include year as a variable to the model.

**Figure 6:**
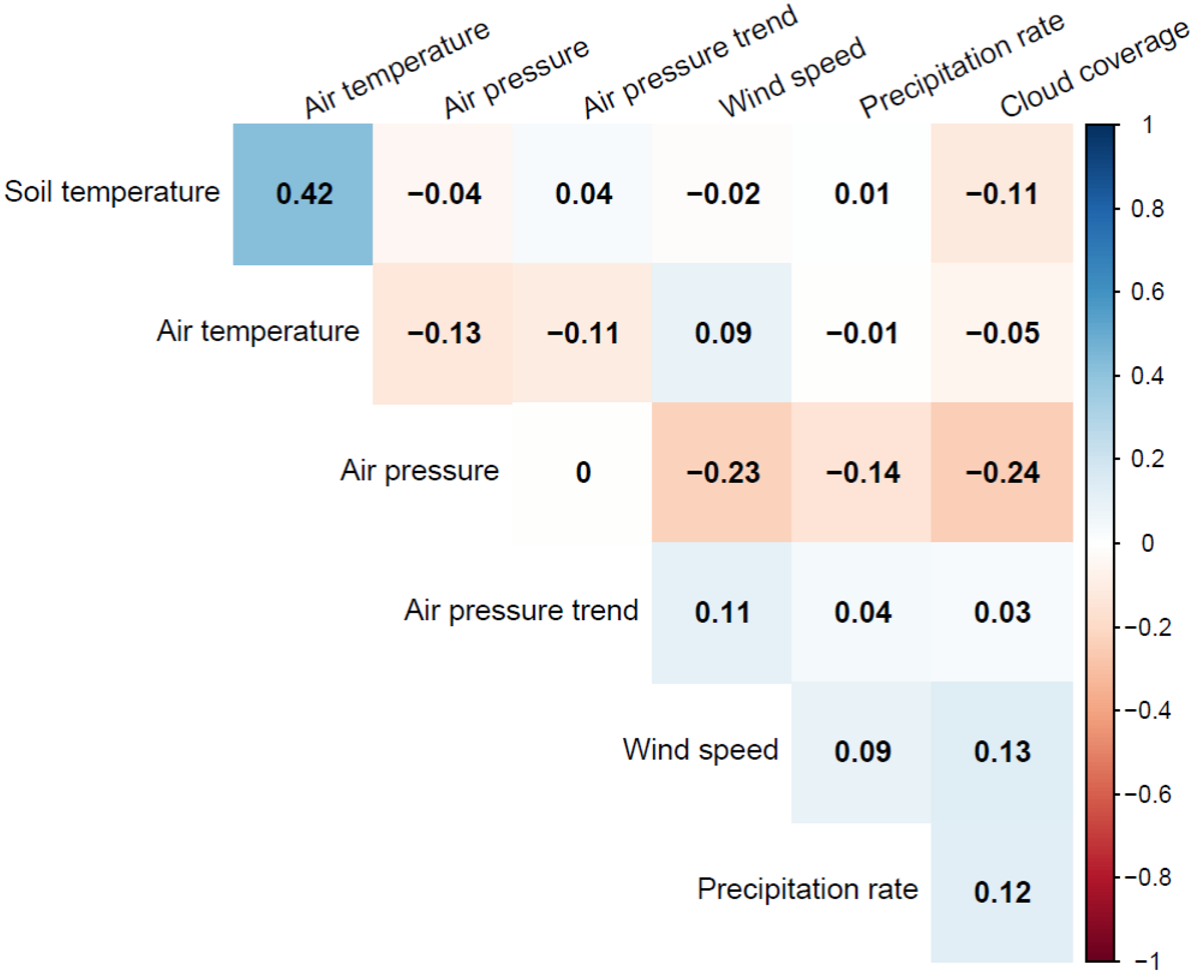
Correlation matrix between environmental predictor variables.

The correlation of *T*_*A*_ and *T*_*S*_ is the highest between any two predictor variables (r = 0.42), showing that collinearity is in general low within our data (see Fig. 6). Excluding either *T*_*A*_ or *T*_*S*_ from the model shows no improvement in explanatory power as measured by the deviance explained (see Table 4). In addition, the standard errors of the estimated coefficients can be considered small in comparison to the coefficients (see Table 1), which suggests against multicollinearity.

**Table 4:** Deviance explained depending on variable exclusion.

Deviance explained	*Myotis myotis*	*Myotis nattereri*	*Myotis daubentonii*	*Myotis bechsteinii*	*Myotis brandtii/ Myotis mystacinus*	*Plecotus auritus/ Plecotus austriacus*
	(GAPM 1)	(GAPM 2)	(GAPM 3)	(GAPM 4)	(GAPM 5)	(GAPM 6)
Full model	0.754	0.848	0.591	0.765	0.845	0.591
Without air temp	0.727	0.828	0.587	0.745	0.837	0.580
Without soil temp	0.753	0.848	0.588	0.765	0.839	0.590

In total, we calculated six different models for each of the six most common species: *Myotis myotis* (all sites), *Myotis nattereri* (all sites), *Myotis daubentonii* (all sites) and *Myotis bechsteinii* (Mayen, Kaub and Neukirchen-Sulzbach). Combined models were estimated for species pairs that are not reliably distinguished from photos: *Myotis mystacinus* /*Myotis brandtii* (Mayen) and *Plecotus auritus/Plecotus austriacus* (all sites). The models were calculated with “R”^76^ and the package “mgcv”^77^ in particular. The code is available upon request through the corresponding author.

## Data Availability

The datasets generated during and/or analyzed during the current study are available from the corresponding author.
